# CRISPR/Cas systems usher in a new era of disease treatment and diagnosis

**DOI:** 10.1186/s43556-022-00095-y

**Published:** 2022-10-14

**Authors:** Ruiting Li, Qin Wang, Kaiqin She, Fang Lu, Yang Yang

**Affiliations:** 1 State Key Laboratory of Biotherapy and Cancer Center, West China Hospital, Sichuan University and Collaborative Innovation Center, Ke-yuan Road 4, No. 1, Gao-peng Street, Chengdu, 610041 Sichuan China; 2 School of Pharmacy, Southwest Minzu University, Chengdu, 610225 Sichuan China; 3 Department of Ophthalmology, West China Hospital, Sichuan University, Chengdu, Sichuan China

**Keywords:** CRISPR, Base editing, Prime editing, Gene therapy, Molecular diagnosis

## Abstract

The discovery and development of the CRISPR/Cas system is a milestone in precise medicine. CRISPR/Cas nucleases, base-editing (BE) and prime-editing (PE) are three genome editing technologies derived from CRISPR/Cas. In recent years, CRISPR-based genome editing technologies have created immense therapeutic potential with safe and efficient viral or non-viral delivery systems. Significant progress has been made in applying genome editing strategies to modify T cells and hematopoietic stem cells (HSCs) *ex vivo* and to treat a wide variety of diseases and disorders *in vivo*. Nevertheless, the clinical translation of this unique technology still faces many challenges, especially targeting, safety and delivery issues, which require further improvement and optimization. In addition, with the outbreak of severe acute respiratory syndrome coronavirus 2 (SARS-CoV-2), CRISPR-based molecular diagnosis has attracted extensive attention. Growing from the specific set of molecular biological discoveries to several active clinical trials, CRISPR/Cas systems offer the opportunity to create a cost-effective, portable and point-of-care diagnosis through nucleic acid screening of diseases. In this review, we describe the development, mechanisms and delivery systems of CRISPR-based genome editing and focus on clinical and preclinical studies of therapeutic CRISPR genome editing in disease treatment as well as its application prospects in therapeutics and molecular detection.

## Introduction

Gene therapy is an approach whereby an “exogenous good gene” is transferred into cells to replace a defective gene in those who suffer from genetic defects [1]. Gene therapy has made remarkable progress in recent years, showing promising clinical results [2]. However, current gene replacement therapy is effective for a part of but precludes utility for other diseases [3, 4]. Genome editing, in contrast, can correct defective DNA in its original location. Therefore, genome editing based on programmable nucleases overcoming the imprecision of current gene therapy is likely to become the next-generation gene therapy technology. At present, there are four major classes of engineered nucleases: meganucleases [5, 6], zinc finger nucleases (ZFNs) [7–9], transcription activator–like effector nucleases (TALENs) [10, 11] and the CRISPR/Cas system [12–14]. Meganucleases, ZFNs and TALENs achieve specific DNA binding via protein-DNA interactions, whereas CRISPR/Cas9 uses simple base-pairing rules between an engineered guide RNA (gRNA) and the target DNA site. CRISPR/Cas9 targets genomic sequences containing protospacer adjacent motifs (PAMs) and complementary to guide RNAs (gRNAs), and generates DSBs [14]. Then, it is taken to the form of either error-prone sequence disruption by non-homologous end joining (NHEJ) or sequence replacement by homology-directed repair (HDR) at the DSB sites to achieve targeted gene disruption, replacement, and modification [15]. CRISPR/Cas9 has been widely used in the research field as well as in disease treatment [16–20]. In recent years, CRISPR/Cas9 has quickly progressed to the clinical stage for the treatment of blood disorders such as β-thalassemia and sickle cell disease, cancer such as metastatic gastrointestinal cancers and metastatic non-small cell lung cancer, eye diseases such as Leber congenital amaurosis (LCA), etc.

To achieve the disease mutation correction and avoid the uncontrolled indel outcomes generated by DSBs, DNA base-editing (BE) and prime-editing (PE) have been developed based on Cas proteins. BEs are capable of C·G to T·A or A·T to G·C conversions by the usage of a catalytically impaired Cas protein to direct an adenine or cytidine deaminase to modify the target window of single-stranded DNA [21]. PEs, the latest addition to the CRISPR genome-engineering toolkit, are composed of a reverse transcriptase (RT) fused to the Cas9 nickase and enable replacement or insertion of any desired sequence based on the information encoded in the co-delivered prime editor guide RNA (pegRNA) [22]. Moreover, PE can mediate not only all 12 base-to-base conversions, but also small insertion and deletion mutations as well [22]. Base editors have been proven to correct the largest single class of human disease-causing mutations, transition mutations, which account for 30% of known disease alleles. While prime editors are more multifunctional, they are capable of installing any base-to-base change inserting up to 44 base pairs and deleting up to 80 base pairs. It suggests that, excluding changes involving aneuploidy, chromosomal rearrangement, sizable duplications, insertions, or deletions, prime editors can, in theory, fix > 89% of known human disease-causing mutations [22–24].

Since its emergence in 2012, CRISPR-based genome editing technology has created immense therapeutic potential. For *ex vivo* research, the modification of T cells and hematopoietic stem cells (HSCs) to treat hematologic disorders [25], viral infections [26] and some refractory cancers [27] are mainly discussed. Since the first autologous CAR therapies targeting CD19 were approved for the treatment of B-cell lymphoma and leukemia in 2019 [28], an increasing number of *ex vivo* studies have come to clinical trials; for example, CTX001 and CRISPR_SCD001 drug products based on modifying HSCs by CRISPR have been created to cure severe sickle cell disease and are currently in phase 3 clinical trials [29]. Meanwhile, in *in vivo* studies, the genome editing technology has also been applied to treat a wide variety of diseases and disorders, mainly liver metabolic disorders [30], ocular disorders [31, 32], and neuromuscular disorders [33, 34], some of which have already been in clinical trials, for example, the CRISPR/Cas9-based EDIT-101 drug product for the treatment of LCA10 [32], the EBT-101 drug product for the treatment of HIV-1 infected adults [26], and the NTLA-2001 drug product for the treatment of hereditary transthyretin amyloidosis [35].

Additionally, due to its capacity for precise gene targeting, the CRISPR system has recently attracted increasing attention as a diagnostic tool. C2c2 (also known as Cas13a), Cas12a and Cas9 are currently widely used in DNA or RNA detection [36]. This diagnostic tool is faster and more sensitive for diagnosing various viruses including SARS-CoV-2 [37], Zika virus [38], human papilloma virus (HPV) [39], Dengue virus [40], Japanese encephalitis virus (JEV) [41], and African swine fever virus [42]. Moreover, the high specificity of this diagnostic tool can also help to discriminate various virus strains [38]. In addition to disease treatment and diagnosis, CRISPR has also been found to hold great potential in synthetic gene circuits, which achieve cell programming programs in a reliable and user-defined manner and detect and could be used to treat multiple tumors [43, 44].

Here, we review the mechanisms, prospects, therapeutic applications, and molecular diagnostic applications of CRISPR genome editing as well as the challenges of the novel technology.

## CRISPR genome editing

### CRISPR/Cas nuclease

**Fig. 1:**
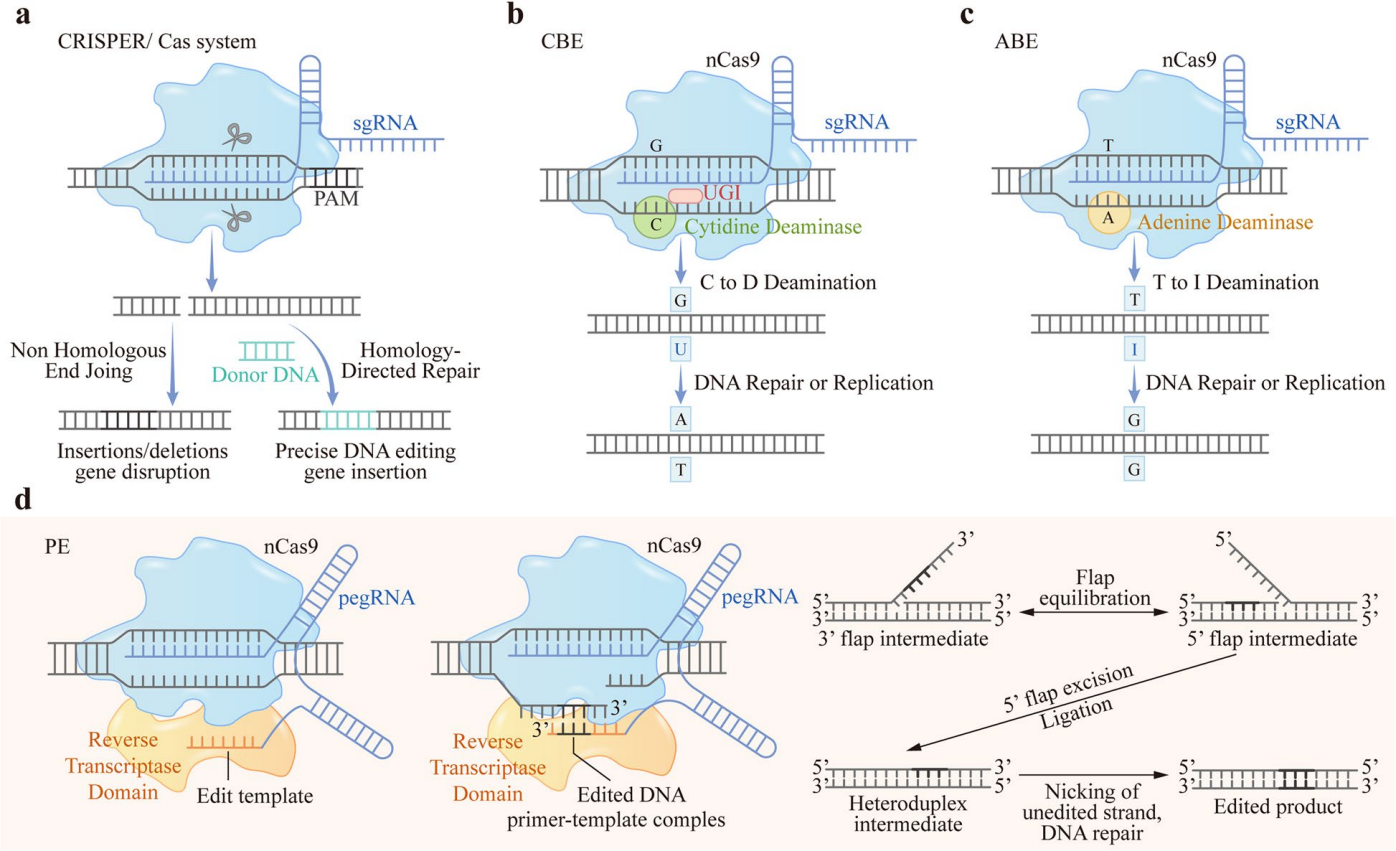
Mechanisms of CRISPR/Cas system, BE and PE. **A** CRISPR/Cas system and NHEJ or HDR mechanisms of DSBs repair. The endonuclease Cas9 is directed to a specific genomic locus and cuts the double stranded DNA, targeted genes can be deleted and repaired via NHEJ. With a donor DNA template, targeted genes can be edited via HDR. **B** The mechanism of CBE. CBE mediates the direct conversion of cytidine to uridine even when located in different sequence motifs, resulting in a C·G to T·A transition. **C** The mechanism of ABE. ABE alters its base pairing preferences by yielding inosine. Inosine prefers to pair with guanosine under specific conditions, resulting in an A·T to G·C transition. **D** The mechanism of PE. PE is composed of a RT fused to Cas9 nickase and a pegRNA, mediates targeted indels and all 12 base-to-base conversions in mammalian cells

CRISPR-Cas RNA-guided nucleases are derived from an adaptive immune system that developed in bacteria to protect them from plasmids and viruses that were invading their environment. Nakata and colleagues identified a cluster of 29 bp repeats downstream of the iap gene located in *Escherichia coli (E. coli)*, found in more than 40% of bacterial species, representing a unique form of clustered repeats in 1987 [45]. Jansen and Mojica referred to these sequences as CRISPR according to their characteristic structure in 2002 [46]. Associated with these repeats are a number of Cas proteins and were classified into three types (types I–III). For types I and III CRISPR, multiple Cas proteins are involved in the recognition and destruction of target genes. The type II system utilizes fewer Cas proteins and works by the function of the single guide RNA (sgRNA) and the single Cas9 endonuclease complex; therefore, it is much simpler to engineer [47–50]. The sgRNA is the combination of the crRNA (CASCADE complex for type I; Cmr or Csm RAMP complexes for type III) and the tracrRNA (transactivating CRISPR RNA) [51]. The Cas9 protein of the Type II CRISPR system is the most widely used for genome engineering due to its precision, and the sgRNA complex interrogates DNA in cells randomly by recognizing the PAM sequence (NGG), a short motif adjacent to the target sequence. Then, the Cas9 protein unwinds the DNA, and the Cas9-associated sgRNA hybridizes with the exposed DNA strand (the protospacer), generating DSBs [50] (Fig. 1a). These breaks are then repaired by the host cell through NHEJ or HDR mechanisms. NHEJ is an efficient but error-prone mechanism that is prevalent and results in small insertions and deletions (indels) at DSB sites. While, HDR is a relatively well-established mechanism with high accuracy during DNA sequence repair [52].

The CRISPR/Cas system represents an efficient genome editing tool after uncontrolled integration into the host genome. Many genome editing methods based on the CRISPR/Cas system for the treatment of diseases have come to clinical trials, and scientists have also developed the CRISPR as an effective tool to detect targeted DNA and RNA. However, the generation of DSBs during the process is still a problem, and more optimized repair mechanisms need to be constructed. Both BE and PE are the newest evolution of CRISPR/Cas-based technologies that correct point mutations in cellular DNA directly without generating DSBs.

### DNA base-editing

DNA BEs comprise a Cas enzyme for binding the targeted DNA sequence and a single-stranded DNA modifying enzyme for altering the targeted nucleotide. Two classes of DNA base-editors have been described thus far: cytosine base-editors (CBEs) convert C·G to T·A [53] and adenine base-editors (ABEs) convert A·T to G·C [54]. Thus, CBEs and ABEs can mediate all four transition mutations of base pairs. In recent years, DNA BEs have also been engineered continually to expand the range of application [55, 56], and a strategy combining CBEs and ABEs has been established to create a dual base-editor system in human cells [57, 58]. To date, CRISPR-guided DNA base editors are widely used for many applications, especially for repairing point mutations [30, 59, 60].

#### Cytosine base-editors

Komor et al. engineered the first-generation cytosine base-editor (CBE1) rAPOBEC1-XTEN-dCas9 by fusing an APOBEC1 cytidine deaminase to dead SpCas9(dCas9) in 2016 [21]. APOBEC1 accepts single-stranded DNA (ssDNA) as a substrate but is incapable of acting on dsDNA, whereas dCas9 contains Asp10Ala and His840Ala mutations that inactivate its nuclease activity. XTEN is a 16 amino acid linker. CBE1 mediates the direct conversion of cytidine to uridine even when located in different sequence motifs and efficiently results in a C·G to T·A transition *in vitro* (Fig. 1b). The deamination efficiency *in vitro* is 25–40%; however, in human cells, it decreases to 0.8–7.7% [21].

To inhibit the conversion and improve base-editing efficiency, a uracil DNA glycosylase inhibitor (UGI) was fused to the C-terminus of BE1 to generate a second-generation cytosine base-editor (CBE2), rAPOBEC1-XTEN-dCas9-UGI. UGI is a small protein from bacteriophages that inhibits BER [61], and BE2 increases base editing efficiency in both bacterial and mammalian cells [53]. Subsequently, the third-generation base editor (BE3) rAPOBEC1-XTEN-nCas9-UGI was designed by turning dCas9 to Cas9 nickase (nCas9). Editing efficiency increases threefold by CBE2 and sixfold further by CBE3, while the indel frequencies of CBE2 and CBE3 are 0.1% and 1.1%, respectively, much lower than indels induced by DSBs [53]. Moreover, Kim et al. used Cas9 variants with different PAM specificities to develop a series of deaminase mutants with varying editing window widths to optimize CBEs in 2017 [62]. To constantly increase the therapeutic applications of gene editing, further optimization of CBEs was still performed [63–67].

#### Adenine base-editors

There are six kinds of pathogenic point mutations in living systems with different frequencies, among which the cytosine deamination rate is close to half, resulting in the C·G to T·A mutation [68–70]. Therefore, a new class of ABEs is required to expand the convert range of CBEs from installing C·G to T·A mutation to convert the A·T base pair back into a G·C base pair (Fig. 1c). ABEs operate a similar mechanism to CBEs; similar to cytosine, adenine alters its base pairing preferences by deaminating the exocyclic amine it contains and then yields inosine. Inosine located in the third position of the tRNA anticodon prefers to pair with A, U, or C in mRNA during translation, but it prefers to match with G when the polymerase active site exists [71].

The major block is that unlike cytosine deaminase, adenosine deaminase acting on ssDNA does not exist in nature. RNA adenosine deaminase is utilized to act on DNA, installed in APOBEC1 of BE3, but no efficiency of adenine base editing was detected [54]. Gaudelli et al. overcame this problem by evolving a tRNA adenosine deaminase of *E. coli* (ecTadA) to manipulate DNA. To obtain the directed evolution and aiming TadA mutants, an antibiotic resistance complementation approach was employed. *E. coli* cells were equipped with TadA mutants and defective antibiotic resistance genes, and the mutant TadA-dCa9 fusion had to correct a deoxyadenosine to a deoxyinosine to realize growth in the presence of antibiotics. The mutant gene of bacteria encoding TadA-dCas9 fusions (TadA*-dCas9) capable of repairing mutant resistance was isolated and then used to develop the first-generation ABEs [54]. The editing rates through simple TadA*-Cas9 nickase fusions are quite low; thus, a single polypeptide chain involving a wild-type noncatalytic TadA monomer, an evolved TadA* monomer and a Cas9 nickase (TadA-TadA*-Cas9 nickase) was designed to optimize ABEs. Moreover, Seventh-generation ABEs were successfully converted from target A-T to G-C (50%) in human cells by extensive protein engineering and controlled evolution [54].

Compared to CBEs, ABEs enable precise conversion of a target A·T to G·C in DNA and yield a much cleaner product (typically ≥ 99.9%) with almost no indels (typically ≤ 0.1%) [54, 72, 73]. Nevertheless, ABEs almost can only match SpCas9, which is different from the broad compatibility of CBEs [74]. To ameliorate this problem, the deaminase component of ABE7.10 was evolved by Richter et al. in 2020 using phage-assisted noncontinuous and continuous evolution (PANCE and PACE)-engineered ABE8e, which increased the function of ABEs and provided higher editing efficiencies when combined with a variety of Cas9 or Cas12 homologs [75].

CBEs and ABEs mediate targeted single-nucleotide conversions without requiring DSBs, minimizing undesired consequences of editing such as indels, large deletions, translocations or other chromosomal abnormalities. On this basis, researchers have focused on developing BEs into a novel therapeutic strategy and have applied BEs to the treatment of some diseases in mice and nonhuman primates [30, 76–85], which we summarized in the last part of this review.

### Prime-editing

CBEs and ABEs can install the four transition mutations (C·G to T·A, A·T to G·C) without DSBs in many cells and organisms, but fail to perform the other eight transversion mutations, such as C·G to G·C, C·G to A·T, A·T to T·A and A·T to C·G [22], which may cause some molecular diseases [86–88]. In 2019, Anzalone et al. described the invention of PE, a gene editing technique that can mediate targeted indels and all 12 base-to-base conversions in mammalian cells, without the need for donor DNA templates or double-strand breaks [22]. PE is composed of an RT fused to Cas9 nickase and a pegRNA. The pegRNA plays a major role in the PE system. On the one hand, by containing the complementary sequence to the target sites that drive nCas9 to its target sequence, it is able to specify the DNA target. On the other hand, it contains an additional sequence enabling to spell the desired sequence changes and bring new genetic information to replace target DNA nucleotides (Fig. 1d). The 5’ end of the pegRNA binds to the primer binding site (PBS) region at the 3′ end of the target DNA strand, exposing the noncomplimentary strand and forming a primer-template complex, while the 3’ end of the pegRNA encodes the desired edit. Upon binding to the target, Cas9 nicks the PAM-containing unbound DNA strand and then primes reverse transcription with the extension in the pegRNA as a template to modify the target region. The reverse transcription template contains the desired DNA sequence changes and the homologous region of the target site to facilitate DNA repair. Subsequently, the edited DNA is newly synthesized with an original DNA sequence containing the 5′ flap without being edited. The 5′ flap is excised, and the 3′ flap is incorporated into the target site by cellular DNA repair processes, generating one edited and one unedited strand of heteroduplex DNA (Fig. 1d). Finally, an additional nick promoted by a simple sgRNA cuts off the unedited strand, resulting in full editing of the dsDNA [89].

Three major versions of the PE system have been developed thus far. PE1 used a wild-type moloney murine leukemia virus (M-MLV) RT fused to a Cas9 nickase and pegRNA, with a maximum editing efficiency of 0.7–5.5%. To optimize PE1, an engineered pentamutant M-MLV RT was used to substitute the wild-type M-MLV RT and created PE2, which increased the editing efficiency approximately threefold. PE3 nicked the unedited strand with an additional sgRNA and enabled 20–50% editing efficiency with 1–10% indels in human HEK293T cells [89, 90]. PEs enable precise targeted indels, and all 12 kinds of point mutations without DSBs or donor DNA templates, result in lower off-target activity, fewer byproducts, and higher editing efficiency. Prime editing is an enormous milestone in the development of gene editing and has an immense potential in clinical applications.

## Delivery systems

**Fig. 2:**
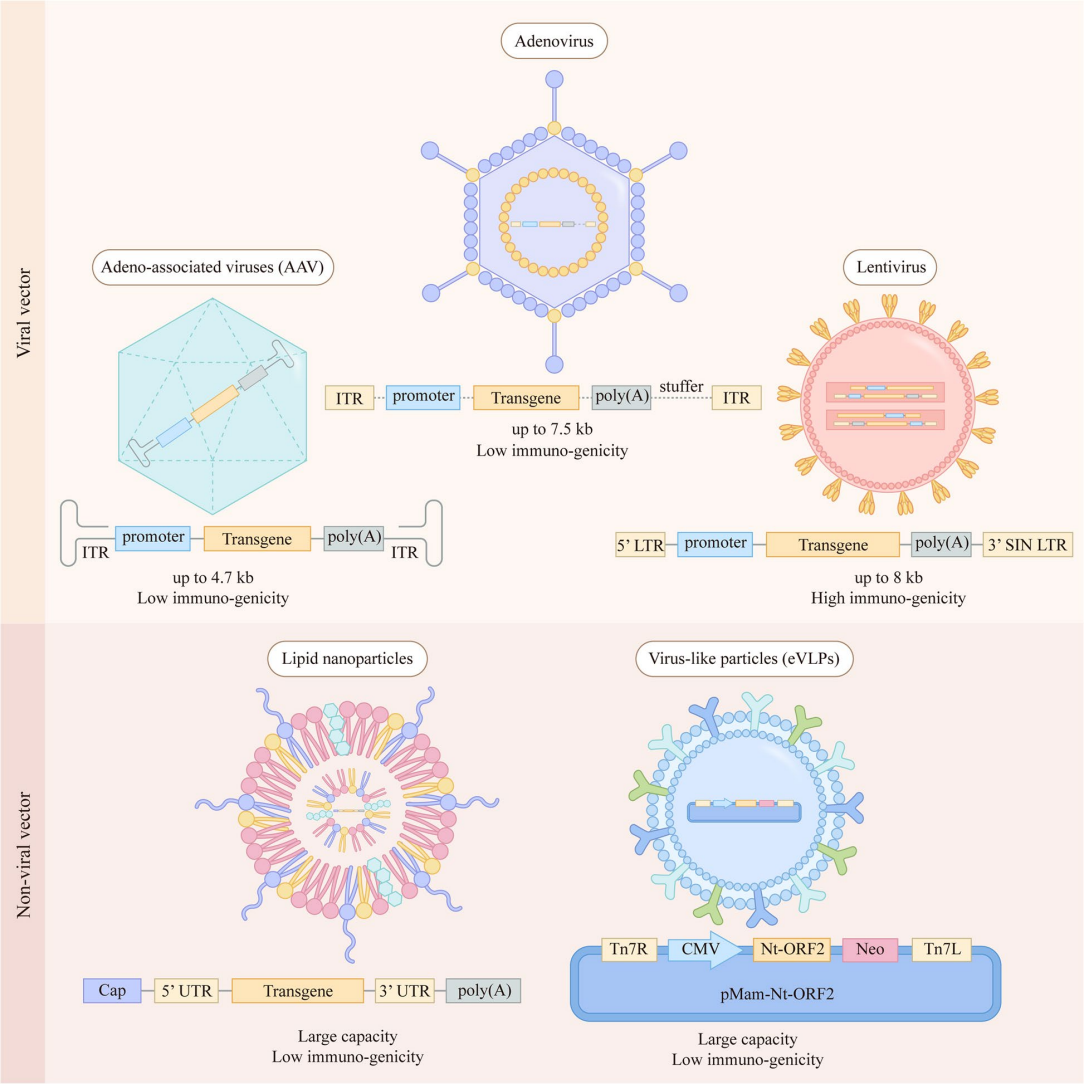
Schematic diagram of viral vectors and non-viral vectors. Schematic of lentivirus and genetic components of lentiviral vectors. Two copies of positive-strand RNA are surrounded by the protein capsid and envelope; the therapeutic transgene is inserted between the two LTRs. Schematic of adenovirus and genetic components of adenoviral vectors. Double strand genome DNA is surrounded by the protein capsid and envelope; the adenoviral vector is stripped of all viral coding sequences, resulting in a vector with only 5’ and 3’ ITRs in addition to a packaging signal. Schematic of AAV and genetic components of rAAV vectors. A single-stranded genome is surrounded by the protein capsid; the therapeutic transgene, along with associated promoter and polyadenylation sequences, is inserted between the two ITRs. Schematic of lipid nanoparticles and genetic components of lipid nanoparticles vectors. The untranslated regions (UTRs) flank both the 5’ and 3’ ORFs, and genes encoding structural proteins are replaced by genes coding for proteins of therapeutic value. Schematic of eVLPs and genetic components of Hepatitis E virus (HEV)-like particles (HEV-LPs) vectors. Hepatitis E virus (HEV) is a liver selective tropism virus in which the major capsid protein of HEV is encoded by its second open reading frame (ORF2) and can be easily assembled to form VLPs17. The ORF2 protein with a deletion of 111 amino acids from the N-terminal end composes the N-terminally-truncated ORF2 (Nt-ORF2), and can form smooth self-assembled HEV-like particles (HEV-LPs)

Efficient and safe gene delivery to target cells and tissues in the human body is one of the most crucial factors and processive challenges for successful therapeutic CRISPR genome editing. First, components including nucleases, the CRISPR/Cas9 systems and the gRNAs need to be delivered efficiently. In addition, the efficiency of homologous recombination, the duration and magnitude of nuclease expression are critical. Moreover, DNA-related cytotoxicity must be low. The present delivery systems of gene editing are classified as viral delivery systems and non-viral delivery systems [91]. There are three major classes of viral delivery systems: adenoviral vector, adeno-associated viral vector (AAV) and lentiviral vector [92] (Fig. 2). Viral gene therapy is an attractive but controversial method in transgenic vectors. It has built successful gene therapy approaches with high delivery efficiencies in multiple disease models *in vivo*, while the limitation of its capacity is still a problem [93]. Non-viral vectors are divided into naked DNA, particle-based and chemical-based vectors [94], and many non-viral gene therapy systems based on liposomes, polymers and nanoparticles have already come into clinical trials [95].

### Viral vector

Adenoviral vectors, AAV and lentiviral vectors are three major classes of viral delivery systems (Fig. 2). Adenoviruses, a group of DNA viruses with double-stranded genomes between 34 and 43 kb, use alternative splicing to encode genes in both sense and antisense directions [96] (Fig. 2). Being an unintegrated virus, it can cause infected differentiated and non-dividing cells to create a significant amount of recombinant virus. Natural adenoviruses have the tendency to transduce pulmonary epithelial cells; thus, human adenoviruses were first used to treat cystic fibrosis [97]. To extend the ability of adenovirus, certain essential viral genes were deleted and replaced with therapeutic elements that express exogenous therapeutic genes, and then the engineered adenovirus vectors used for gene therapy, vaccines, and cancer therapy were obtained [98]. Adenovirus vectors are currently the most commonly employed vectors for cancer gene therapy, for instance, the use of adenoviruses targeting the SERPINA1 gene in hepatocytes to rescue the pathological liver phenotype in a mouse model of α1-antitrypsin deficiency [99].

AAV is a small and nonpathogenic parvovirus composed of a 4.7 kb ssDNA genome within a nonenveloped icosahedral capsid. The wild-type AAV belongs to the *Parvoviridae* family and is not able to automatically replicate unless the adenovirus exists [100]. AAV can infect mammals but remains inactive without integrating into the genome of the host and thus has no genotoxic effect. Furthermore, although a small portion of humans are AAV seropositive, AAV capsids have a lower systemic inflammatory response than adenoviruses [101, 102]. The genome of AAV contains three open reading frames (ORFs) (*rep*, *cap*, *aap*) flanked by ITRs. In recombinant AAV vectors, A therapeutic transgene linked to promoter and polyadenylation sequences is placed between the viral ITRs in place of *rep* and *cap* (Fig. 2). The AAV variants are abundant; there are 11 natural serotypes and more than 100 variants of engineered AAV with diverse amino acid sequences and gene delivery properties [103]. AAV was first adapted as a mammalian DNA cloning vector almost 40 years ago [104] and benefited from the non-genotoxicity and low immunogenicity. AAV has gradually been considered as the most promising method for gene therapy delivery systems. Glybera, the first gene therapy product approved in Europe for patients with lipoprotein lipase deficiency in 2012, was based on an AAV gene delivery system [105–107]. In recent years, various AAV vector-mediated gene therapies have produced clinical benefits, for example, the treatment of various eye diseases [108, 109] and spinal muscular atrophy [100, 110], as well as some rare diseases, including hemophilia and Duchenne muscular dystrophy [111–113]. In fact, further modification is also required to optimize the capacity, transduction efficiency, and immune response of AAV to facilitate the success of AAV gene therapy.

Lentiviruses are a subclass of retroviruses based on HIV and other nonhuman lentiviruses, which are single-stranded RNA (ssRNA) viruses and can integrate viral DNA into the genome of targeted cells. Replication of retroviruses has a deontic step: RNA copies into DNA and then integrates into the genome of the host cell. Two copies of positive-strand RNA with three genes are packaged by lentiviruses: *gag* (encoding structural proteins), *pol* (encoding RT, integrase, and protease enzymes that are packaged with the RNA strands inside the virus), and *env* (encoding envelope proteins that coat the virus), while accessory protein genes are flanked by the long terminal repeat (LTR) that also functions as a promoter sequence (Fig. 2). As vectors, lentiviral vectors (LVs) are capable of delivering transgenes up to 8 kb in size and transducing dividing as well as non-dividing cells such as neurons, hematopoietic stem cells and T cells. LVs represent a major vector for the treatment of monogenic diseases and adoptive cell therapy trials where gene delivery is required [114]. For genetic components of LVs, the therapeutic transgene is inserted between the viral LTRs, and the three genes *gag*, *pol*, and *env* are the essential elements of the production of LVs [115]. Retroviruses were the only practicable method to modify patients’ genes before the discovery of CRISPR-Cas systems [101]. In the early 1990s, the first clinical trial of gene therapy for genetic diseases was started using retroviral-mediated transfer of the adenosine deaminase gene into T cells to cure the severe combined immunodeficiency caused by the lack of adenosine-deaminase [116, 117]. Naldini et al. created an *in vivo* lentiviral gene delivery system and achieved stable transduction of nondividing cells through LVs in 1996 [118]. Currently, LVs are widely used in laboratory and clinical gene therapy applications [119–121], especially most used for *ex vivo* gene transduction, due to their capacity to integrate transgenes into the genome of the host cell and to infect both proliferating and nondividing cells [122], with relatively large packaging capacity and low immunogenicity [123].

### Non-viral delivery system

Using a viral delivery system to deliver therapeutic components is the most widely used approach thus far. However, it also brings some risks including increasing the frequency of off-target editing [124] and relatively increasing the possibility of oncogenesis caused by the integration of viral vectors into the genome of targeted cells [92]. Compared to viral delivery systems, non-viral delivery systems have less gene delivery efficiency, but have lower immune responses, less insertional mutagenesis, greater capacity and lower costs [94, 95, 125]. As is known, the non-viral vectors are divided into naked DNA, particle-based and chemical-based vectors [94]. Due to the advantages of the non-viral delivery system, a large number of research efforts and advancements have brought nanoparticle-based, lipid-based and polyplexes-based non-viral gene delivery vectors into the clinic [95].

Liposomes are spherical delivery systems with hydrophilic polar head groups and hydrophobic tails that are effective at encasing both water-soluble and water-insoluble substances within their hydrophilic core and lipid membrane, respectively [126]. Lipid nanoparticles (LNPs) were explored in 1999 using stabilized plasmid lipid particles through a detergent dialysis method [127], which are quite different from classical liposomes, especially LNPs that do not display a lipid bilayer surrounding the aqueous core [128]. In 2018, Onpattro, the production of LNPs for the treatment of amyloidosis was approved in the US and EU, which confirmed its ability to deliver nucleic acid drugs [129]. A delivery system containing cationic polymers has been approved, which has the advantages of formulating smaller uniform particle sizes and improving transfection efficiency [130].

Recently, a novel non-viral delivery system named engineered virus-like particle (eVLPs) vector was created by David Liu's group [131]. The VLP vector is composed of infectious viral proteins but lacks viral genetic material, and has been engineered to efficiently deliver therapeutic protein RNPs including BEs and Cas9 nuclease, *in vivo*. The efficient packaging and delivery of RNPs overcome the problems of cargo packaging, release, and localization. Moreover, compared with the longer time that DNA is present in target cells, the existence of RNP is quite short, which reduces the frequencies of off-target editing.

## Disease treatment

**Fig. 3:**
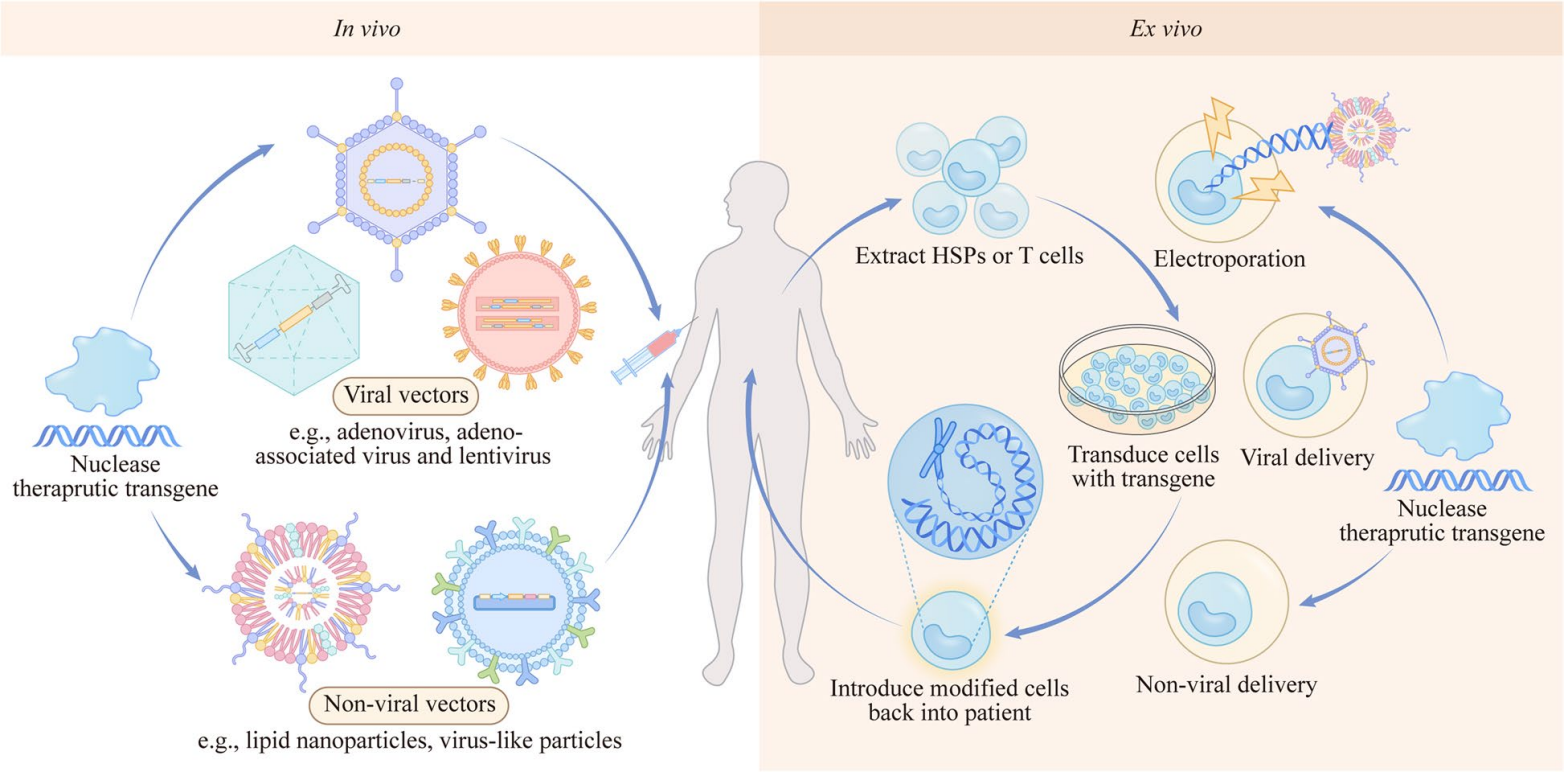
*Ex vivo* and *in vivo* strategies for therapeutic CRISPR genome editing. *Ex vivo* and *in vivo* strategies for therapeutic CRISPR genome editing. For *ex vivo* strategies, HSPs or T cells are extracted from patients, deliver the nuclease and therapeutic transgene to the cells by electroporation, viral vector or non-viral vector, then transduce cells with transgene and introduce modified cells back into patients

CRISPR-based genome editing is able to precisely modify any genomic sequence, and this feature creates immense therapeutic potential. Next, we focus on the ongoing clinical strategies using both *ex vivo* and *in vivo* strategies for major categories of disease treatment by therapeutic CRISPR gene editing (Fig. 3).

### *Ex vivo* gene editing

The strategy of altering genes in autologous cells *ex vivo* is the most straightforward application of gene editing (Table 1). In this process, somatic cells are isolated first, modified by gene editing tools, and finally delivered back to the patients’ organs (Fig. 3).

**Table 1:** Reported *ex vivo* clinical trials of CRISPR-based therapeutic gene editing

Identifier	Phase	Disease	CRISPR system	Modified gene/cell	Delievry approach
**HSCs**					
NCT03655678	Phase 1/2/3	Transfusion-Dependent β-Thalassemia	CRISPR/Cas9	BCL11A enhancer	Electroporation
NCT03745287	Phase 1/2/3	Sickle Cell Disease	CRISPR/Cas9	BCL11A enhancer	Electroporation
NCT04774536	Phase 1/2	Sickle Cell Disease	CRISPR/Cas9	HPSCs	-
NCT04819841	Phase 1/2	Sickle Cell Disease	CRISPR/Cas9	HbS	-
NCT04925206	Phase 1	Transfusion Dependent β-Thalassaemia	CRISPR/Cas9	HSCs	-
**T cell immunotherapy**					
NCT04426669	Phase 2	Metastatic Gastrointestinal Cancer	CRISPR/Cas9	CISH	Electroporation
NCT04560790	Phase 2	Viral Keratitis	CRISPR/Cas9	HSV-1	-
NCT03398967	Phase 2	Leukemia and Lymphoma	CAR-T	T Cell	-
NCT03166878	Phase 2	Leukemia and Lymphoma	CAR and CRISPR RNA	TCR and B2M	Lentiviral and electroporation
NCT04990557	Phase 2	COVID-19	CRISPR/Cas9	PD-1 and ACE2	-
NCT03044743	Phase 2	Epstein-Barr Virus	CRISPR/Cas9	PDCD1	Electroporation
NCT03399448	Phase 1	cystic fibrosis, refractory cancer	CRISPR/Cas9	TCRα, TCRβ and PD-1	Electroporation
NCT04035434	Phase 1	B Cell Malignancies	CRISPR/Cas9	T cell	-
NCT04557436	Phase 1	Lymphoblastic Leukemia	CRISPR/Cas9	CD52 and TRAC	Lentiviral
NCT02793856	Phase 1	Non-small Cell Lung Cancer	CRISPR/Cas9	PDCD1	Electroporation
NCT04037566	Phase 1	Leukemia or Lymphoma	CRISPR/Cas9	HPK1	Electroporation
NCT03545815	Phase 1	Mesothelin Positive Multiple Solid Tumors	CRISPR/Cas9	PD-1 and TCR	-
NCT04637763	Phase 1	B Cell Non-Hodgkin Lymphoma	CRISPR	T cell	-
NCT05037669	Phase 1	Leukemia and Lymphoma	CRISPR/Cas9	CIITA and TCR-α	Electroporation
NCT04244656	Phase 1	Multiple Myeloma	CRISPR/Cas9	T cell	-
NCT04438083	Phase 1	Renal Cell Carcinoma	CRISPR/Cas9	T cell	-
NCT03747965	Phase 1	Mesothelin Positive Multiple Solid Tumors	CRISPR/Cas9	PD-1	-
NCT04035434	Phase 1	B-Cell Malignancies	CRISPR/Cas9	T cell	-
NCT04502446	Phase 1	T or B Cell Malignancies	CRISPR/Cas9	T cell	-
NCT04417764	Phase 1	Hepatocellular Carcinoma	CRISPR/Cas9	PD-1	-
**pancreatic endoderm cells**					
NCT05210530	Phase 1	Diabetes Mellitus	CRISPR/Cas9	pancreatic endoderm cells	-

^¶^ Data were obtained from ClinicalTrials.gov and PubMed

#### Gene editing of hematopoietic stem cells

Gene therapy of autologous hematopoietic stem cells (HSCs) with integrating vectors has the potential to cure many inherited disorders, especially diseases of the blood system and immune deficiencies. The first clinical application of HSC gene therapy was applied to the most severe immunological defects called primary immunodeficiencies (PIDs). PIDs are a large group of diseases, and four of the most extensively studied PIDs are X-linked severe combined immunodeficiency (SCID-X), adenosine deaminase deficiency (ADA-SCID), Wiskott-Aldrich syndrome (WAS), and chronic granulomatous disease (CGD). The hematopoietic system of PIDs is intrinsic, making them the ideal target for allogeneic hematopoietic stem cell transplantation (alloHSCT) [132]. With the discovery of retroviruses, the first HSC gene therapy trials for SCID-X1 and ADA-SCID were conducted [133–136]. Then, the CRISPR/Cas9 system was used to repair mutations in the CYBB gene of CD34^+^ HSCs from patients with the immunodeficiency disorder CGD [137, 138]. In addition, with the help of AAV, the CRISPR/Cas9 system also achieved homologous recombination of the β-globin gene in HSCs to cure red blood cell disorders [139]. In recent years, CRISPR/Cas9-based CRISPR_SCD001 and GPH101 drug products have been undergoing clinical trials (ClinicalTrials.gov Identifier: NCT04774536, CT04819841). They are CRISPR/Cas9 edited red blood cells designed for a single infusion of differentiated CD34 + hematopoietic stem cells (HSPCs) modified by the sickle allele in patients with severe sickle cell disease (SCD), and are currently evaluating HSCT safety and efficacy.

#### Gene editing of T cells

Another advanced *ex vivo* gene editing strategy is the modification of T cells, especially the treatment of primary HIV infection by knocking out the CCR5 coreceptor. In an earlier study, researchers knocked out the CCR5 gene of T cells via ZFNs and then engrafted the corrected T cells into HIV-infected mice, successfully decreasing viral loads and increasing CD4^+^ T cell numbers [140]. In addition to ZFNs, similar gene-editing strategies to knock out CCR5 with TALENs [141, 142], CRISPR/Cas9 [26, 142, 143], and meganucleases [144] have been developed recently. In 2020, three patients with refractory cancer participated in the first-in-human phase 1 clinical trial testing the viability and safety of multiplex CRISPR/Cas9 editing to generate T cells, and patients performed to improve antitumor immunity within the modified T cells persisted for up to 9 months (ClinicalTrials.gov Identifier: NCT03399448) [145].

Furthermore, *ex vivo* T cells modified to express chimeric antigen receptors (CAR-T) and recombinant T-cell receptors (rTCRαβ) are feasible for cancer immunotherapy [146]. In 2019, the first autologous CAR product targeting CD19 obtained approval for marketing to treat B-cell-derived lymphoma and leukemia [28]. However, a subset of patients who accepted CD19-directed CAR-T-cell therapy for the treatment of relapsed or refractory B-cell malignancies suffered the relapse due to the loss of CD19 in tumor cells or were unable to receive this highly active therapy because of failed expansion. Moreover, infantile cancer patients have a small blood volume, and it is a challenge to manufacture an effective therapeutic product for them. Personalized autologous T cell manufacturing is the inherent characteristic of autologous CAR-T cell therapy, resulting in the difficulty of industrialization of autologous CAR-T cell therapy. One of the improved methods is using dual specificity CD19 and CD20 or CD22 CAR-T cells to recognize and kill CD19-negative malignant cells by recognizing CD20 or CD22 and then achieve immunotherapy for relapsed or refractory leukemia and lymphoma (ClinicalTrials.gov Identifier: NCT03398967).

Another optimized method is to combine the CAR-T and CRISPR mRNA electroporation to disrupt endogenous TCR and B2M genes simultaneously by LVs. This method generates universal CD19-specific CAR-T cells (UCART019) derived from one or more healthy unrelated donors without graft-versus-host-disease (GVHD) and minimizes their immunogenicity; currently, it is in progress of a phase 2 clinical trial (ClinicalTrials.gov Identifier: NCT03166878). Additionally, the NTLA-5001 as a T-cell receptor engineered T (TCR-T) cells directed drug based on CRISPR/Cas9 was also assessed in a clinical trial to investigate the safety and efficacy in subjects with acute myeloid leukemia (ClinicalTrials.gov Identifier: NCT05066165).

To date, there have been various clinical trials using CRISPR systems to modify human T cells and have a great chance of curing many refractory diseases. For example, CRISPR/Cas9 mediated PD-1 knockout T cells from autologous origin for advanced Epstein-Barr virus (EBV)-associated malignancies (ClinicalTrials.gov Identifier: NCT03044743), genetically engineered T cell therapy for solid tumors in the setting of novel checkpoint inhibition to treat metastatic gastrointestinal cancers (ClinicalTrials.gov Identifier: NCT04426669), the BD111 drug product for the treatment of refractory viral keratitis (ClinicalTrials.gov Identifier: NCT04560790) and so on.

COVID-19 is a worldwide pandemic. It has been reported that in patients infected with SARS-CoV-2, the PD-1 and Tim-3 expression on the surface of T cells was increased significantly, which was directly related to the disease's severity and was also increased in other viral infections. Using CRISPR/Cas9 to knock out PD-1 and ACE2 to modify human T cells and achieve the induction of long-term immunity in COVID-19 patients is a potential and effective method to cure the infectious disease. In this clinical trial, exhausted virus-reactive CD8^+^ memory T cells will be collected and both the programmed cell death protein 1(PDCD1) gene and ACE2 gene will be knocked out by CRISPR/Cas9 in the laboratory. Then the lymphocytes will be selected and expanded *ex vivo*, and reinfused into patients (ClinicalTrials.gov Identifier: NCT04990557).

In addition to gene editing of T cells, some other clinical trials are also underway utilizing CRISPR/Cas9 with AAV vectors or LVs as the delivery system to modify B cells for the treatment of refractory B cell malignancies (ClinicalTrials.gov Identifier: NCT04035434, NCT04557436) (Table 1).

### *In vivo* gene editing

Gene editing *in vivo* is a large-extended strategy for targeted gene correction in tissues, as cell transplantation presents obstacles under certain conditions. To achieve gene editing *in vivo*, the effective delivery of gene-editing nucleases and donor vectors to target tissues, low off-target frequencies, and low genotoxic effects are all required (Fig. 3). Initially, highly effective nuclease-mediated gene editing *in vivo* was demonstrated in a study, that delivered ZFNs and a factor IX cDNA to the liver of a promoter-free animal model of hemophilia B by AAV vector [147]. Later, an increasing number of *in vivo* gene editing studies with various therapeutic strategies for multiple diseases were conducted, especially CRISPR-based genome editing. Here, therapeutic *in vivo* gene editing is discussed by the classification of tissues with representative diseases (Table 2).

**Table 2:** Reported *in vivo* clinical trials of CRISPR-based therapeutic gene editing

Identifier	Phase	Disease	CRISPR system	Modified gene	Delivery approach
NCT03872479	Phase 2	LCA10	CRISPR/Cas9	CEP290	AAV
NCT03057912	Phase 1	HPV-related Cervical Intraepithelial Neoplasia I	CRISPR/Cas9	HPV16-E7	-
NCT05143307	Phase 1	HIV-1	CRISPR/Cas9	-	AAV
NCT05144386	Phase 1	HIV-1	CRISPR/Cas9	-	AAV
NCT04601051	Phase 1	ATTRv-PN, ATTR-CM	CRISPR/Cas9	TTR	LNPs

^¶^ Data were obtained from ClinicalTrials.gov and PubMed

#### Liver-targeted gene editing

Many different types of liver diseases have the potential to be treated by gene correction, including metabolic liver diseases, viral hepatitis and hepatocellular carcinoma (HCC). Metabolic liver diseases mainly include clotting disorders (hemophilia A and hemophilia B), hereditary tyrosinemia, lysosomal storage disorders (Fabry disease, Gaucher disease, Pompe disease, von Gierke disease), and ornithine transcarbamylase deficiency (OTCD) (Table 2). Among them, hemophilia is caused by factor VIII (FVIII) or factor IX (FIX) mutations in the clotting factor genes, which disrupt the clotting pathway. Metabolic liver diseases are among the best candidates for genome editing therapeutic strategies, as many of them are too severe to be treated with drugs and require orthotopic liver transplantations. Gene editing has become a potential method to correct the metabolic liver disease phenotype, some of which have obtained significant effects [148, 149]. Since 2011, nuclease-mediated gene editing *in vivo* has been used to ameliorate hemophilia B in infants by delivering ZFNs and a factor IX cDNA using AAV vectors [147] and was later demonstrated to be effective in adult mice [150]. In addition to ZFNs, CRISPR/Cas9-mediated gene correction was created and later ameliorated hemophilia in mice [151, 152].

Another metabolic genetic disorder named hereditary tyrosinemia type I (HTI) is caused by a mutation in fumarylacetoacetate hydrolase (FAH), resulting in toxic metabolite accumulation. In 2014, CRISPR/Cas9 was used for the first time to correct the HTI in a mouse model following hydrodynamic tail vein injection of plasmid DNA, which allowed the corrected cells to repopulate the liver successfully [153]. Later, therapeutic strategies based on CRISPR/Cas9 were optimized continuously. In 2016, instead of editing the disease-causing gene, a disease-associated pathway gene named *Hpd* was deleted using CRISPR/Cas9 and successfully rerouted tyrosine catabolism in some mice [149]. In the same year, using LNP-mediated delivery of Cas9 mRNA with AAV encoding a sgRNA and a repair template to induce the generation of FAH-positive hepatocytes by correcting the causative Fah-splicing mutation also successfully redeemed disease symptoms such as weight loss and liver damage [154]. Later, an improved Cas9n was engineered to reduce the numerous undesired mutations caused by wild-type Cas9, and the Cas9n-mediated genome editing in treating HTI suggested a safer and optimized therapeutic CRISPR genome editing strategy [148]. Recently, BEs were constructed and greatly extended the CRISPR/Cas9 system; the plasmid DNA encoding the ABE and sgRNA corrected an A > G splice-site mutation, and this treatment successfully relieved the symptoms of HTI [84]. Researchers also used BE to correct genetic point mutations by AAV in neonatal phenylketonuria (PKU) mice in 2022 [30, 85]. LNP-based delivery of mRNA encoding ABE and sgRNA targeting PCSK9 has been proven to reduce the blood low-density lipoprotein (LDL) levels efficiently and safely [81]. As a novel gene editing tool, BE has great therapeutic potential. Other liver metabolism diseases including lysosomal storage disorders and OTCD have also been successfully treated in animal models by *in vivo* gene editing [155, 156] (Table 2).

Hepatitis B virus (HBV) is a type of viral hepatitis, and its high infection rate makes it one of the most affected diseases in the world. Vaccines have been the most effective prevention and treatment strategy until now but can only inhibit HBV replication while cannot eliminate the covalently closed circular DNA (cccDNA) of HBV still carried in the hepatocyte nucleus [157, 158]. Therefore, using CRISPR/Cas9 to specifically target and disrupt the cccDNA of HBV has become an attractive and novel strategy to cure chronic hepatitis B [158–161]. In 2014, Lin *et al*. designed a CRISPR/Cas9 system with HBV-specific gRNAs that significantly decreased the production of HBV core and surface proteins in Huh-7 cells transfected with an HBV expression vector in the mouse model [159]. Furthermore, targeting the ORFs S and X of HBV by CRISPR/Cas9 reduced the serum surface-antigen levels and inactivated HBV in chronically and *de novo *infected cells to some extent [162, 163]. DNA polymerase κ (POLK), a Y-family DNA polymerase that significantly contributes to the formation of cccDNA during *de novo* HBV infection, is most active in non-dividing cells. Thus, the expression of POLK in HepG2-NTCP cells can be depleted by siRNA and CRISPR/Cas9 to inhibit the conversion of rcDNA into cccDNA [164]. Recently, after the SaCas9 and S gene targeting gRNA were introduced into HepG2.2.15 cells by single stranded adeno-associated viral vectors (ssAAVs), targeted mutation of HBV DNA was observed, indicating that the inactivation of cccDNA was successful [160]. Hepatitis C virus (HCV) is another kind of widespread chronic hepatitis. A study showed that Francisella novicida Cas9 endonuclease (FnCas9) directed by an engineered gRNA is capable of inhibiting HCV within eukaryotic cells [165].

The ability to precisely mediate gene KO in numerous cell types makes CRISPR/Cas9 a potential technique for the treatment of multiple cancers, such as HCC. CRISPR/Cas9 can precisely target many tumor-associated genes, including the tumor-promoting genes G9a, ASPH, eEF2, NCOA5, CXCR4 and CDK7, and the tumor-suppressing genes p53 and PTEN [166–171]. G9a is an important epigenetic regulator. As a histone methyltransferase, it is associated with the occurrence and development of human hepatocellular carcinoma. The poor prognosis in HCC is indicated by the upregulation of G9a. Inactivation of G9a by RNAi knockdown and CRISPR/Cas9 knockout can suppress the progression of HCC cells *in vitro* and inhibit HCC tumorigenicity *in vivo* [166]. ASPH, aspartate β-hydroxylase, an enzyme involved in the malignant transformation process, is overexpressed in HCC tumors. ASPH knockout was achieved by the CRISPR/Cas9 system and decreased HCC growth and progression, suggesting that ASPH enzymatic activity is a novel therapeutic target mediated by CRISPR/Cas9 for HCC [170]. eEF2, eukaryotic elongation factor 2, is a prognostic marker which kinase is a potential therapeutic target in HCC. Compared with non-tumorous tissue, the activity of the regulating eEF2 kinase in tumors is more than four times higher, while proliferation and growth are decreased in CRISPR/Cas9-mediated eEF2 kinase knockout HCC cells [167]. NCOA5, a nuclear receptor coactivator, performs critical roles in the emergence of numerous cancers, and CRISPR/Cas9 deletion of NCOA5 reduces hepatocellular carcinoma cell migration and proliferation by preventing the epithelial-to-mesenchymal transition [168]. CXCR4, CXC chemokine receptor 4, is linked to poor clinical outcomes and a decreased survival rate in HCC. Utilizing the CRISPR/Cas9 system to mediate genome engineering of CXCR4 can decrease the malignancy of hepatocellular carcinoma cells *in vitro* and *in vivo* [169]. CDKs, cyclin-dependent kinases, regulate the gene transcription of HCCA, and a CRISPR screen identified CDK7 as a therapeutic target for HCC [171].

#### Ocular disorders

Therapeutic CRISPR/Cas genome editing technology has been rapidly developed in treatments of ocular disorders since its discovery in 2012, and now the EDIT-101 drug product based on CRISPR/Cas system with SaCas9 gRNAs has been developed for the treatment of LCA10 [32] (ClinicalTrials.gov Identifier: NCT03872479). The LCA, retinitis pigmentosa (RP), proliferative diabetic retinopathy (PDR), wet age-related macular degeneration (wAMD), corneal dystrophy (CD) and optic nerve (ON) diseases are six classes of ocular disorders that have advantages in gene editing treatments.

LCA is a part of the spectrum of early-onset retinal dystrophy (EORD). LCA10 is the most prevalent subtype of LCA, a severe retinal degeneration caused by mutations in the CEP290 gene. Gene therapy clinical trials for treating LCA2 by subretinal injection of AAV encoding the full RPE65 gene have shown great success in terms of both safety and efficiency [172, 173]. However, the large size of the CEP290 gene limits the loading capacity of the full length gene, and the CRISPR/Cas9 system has been developed to optimize the strategy [174]. A therapeutic genome-editing candidate EDIT-101 with SaCas9 gRNAs has also been developed for the treatment of LCA10 [32] (ClinicalTrials.gov Identifier: NCT03872479). In 2019, Boye's group finished the first *in vivo* CRISPR genome editing in the retina of the nonhuman primate macaque. SaCas9 delivered by AAV5, together with a sgRNA targeting GUCY2D, reduced the expression of retinal guanylate cyclase-1 (retGC1) and improved the retinal function and structure. GUCY2D is the gene encoding retGC1, and mutations in this gene lead to autosomal dominant cone-rod dystrophy (CORD6) and cause LCA1 [175]. CBEs and ABEs can correct point mutations precisely, and the subretinal injection of an LV expressing an ABE and a sgRNA targeting the *de novo* nonsense mutation in the Rpe65 gene can correct the pathogenic mutation with an efficiency of 29%. The formation of indel and off-target mutations minimally restores RPE65 expression and improves the retina in many aspects in ABE-treated mice [82].

In addition to LCA, RP is another large category of ocular disorders caused by the mutation of the rhodopsin (RHO), NRL, Pde6b or other genes, leading to rod photoreceptor degeneration that invariably causes vision loss. P23H is the most common mutation in the RHO gene. To inactivate the RHO-P23H mutant, an AAV9-based CRISPR/Cas9 delivery strategy was used, and the phenotypes and functions of the retina were successfully improved [31]. In the same year, researchers utilized both SpCas9 variants and truncated sgRNAs to discriminate a single-nucleotide mutation in RHO-P23H mice, and in treated areas of the RHO-P23H retina at 5 weeks of age, the rate of photoreceptor cell degeneration in the outer nuclear layer was significantly delayed [176]. Nevertheless, further optimization is still required because not every mutation in the RHO gene can find a proper CRISPR design; thus, it is necessary to develop a novel gene editing strategy to overcome the genetic heterogeneity in RP resulting from mutations in RHO. An optimized experimental study combining both gene ablation and gene replacement destroys the expression of endogenous RHO gene in a mutation-independent manner *via* an improved CRISPR-based gene deletion delivered by AAV. The expression of wild-type protein was restored *via* exogenous cDNA, and the thickness of the outer nuclear layer and the results of electroretinography improved significantly after the subretinal injection of combination ablate-and-replace gene therapy [177]. The NRL gene encodes neural retina-specific leucine zipper protein, which determines the photoreceptor development and is associated with RP. Using AAV-mediated CRISPR/Cas9 delivery to postmitotic photoreceptors to target and disrupt NRL in rods, following the treatment, rods gained partial features of cones and presented with improved survival in the presence of mutations in rod-specific genes, consequently preventing secondary cone degeneration [178, 179]. In another study, instead of disrupting the NRL gene for the transformation of rods to cone-like cells, a CRISPRi technique was adopted to repress NRL gene expression, and downregulation of NRL in the Rd10 mouse photoreceptors was achieved *in vivo*. The CRISPRi system includes a dCas9 that is fused with a gene repressor protein such as KRAB, the dCas9/repressor complex results in sequence-specific gene repression with the guidance of sgRNA [180]. Mutations in the Pde6b gene also result in RP, and the Pde6b gene regulates intracellular cGMP levels. Researchers used CRISPR/Cas genome editing to untangle the effects of two potentially pathogenic genetic differences [181] and attempted to repair causative mutations in a preclinical model of RP [31, 182].

Ocular angiogenesis is associated with a variety of human diseases, including PDR and AMD, in which vascular endothelial growth factor receptor 2 (VEGFR2) plays an essential role. Using the CRISPR/Cas9 system with AAV to deplete VEGFR2 in vascular endothelial cells (ECs) provided more opportunities to suppress angiogenesis in mouse models of oxygen-induced retinopathy and laser-induced choroid neovascularization [183, 184] (Table 3). Specifically, using AAV9-delivered CjCas9 to target the VEGFA or HIFLA gene in RPE cells can reduce the size of laser-induced choroidal neovascularization, indicating that *in vivo* CjCas9-based genome editing is useful for the treatment of wAMD [185]. LV-delivered CRISPR is also able to disrupt the VEGFA gene efficiently [186].

**Table 3:** Representative preclinical studies of therapeutic gene editing

Target	Gene editing tool	Modified gene	Delievry approach	References
**Hematologic disorders**				
SCID-X	ZFNs	IL2RG	-	[9]
	ZFNs; donor DNA template	IL2RG	Electroporation; LV	[187]
X-CGD	CRISPR/Cas9	CYBB	Electroporation	[137, 138]
Sickle cell disease and β-thalessemia	CRISPR/Cas9	β-globin	AAV	[139]
	ZFNs	β-globin	Adenovirus	[188, 189]
	CRISPR/Cas9	HBB	Electroporation	[190, 191]
**Viral infections**				
HIV	ZFN; TALENs; CRISPR/Cas9; meganuclease	CCR5	Adenovirus, lentiviral,	[26, 140–143]
**Liver-targeted gene editing**				
Hemophilia	ZFNs	F9	AAV	[147, 150]
	CRISPR/Cas9	F9	Adenovirus	[151, 152]
Hereditary	CRISPR/Cas9	FAH	Adenovirus	[148, 153]
tyrosinemia type I	ABEs	FAH	LNPs	[84]
OTCD	ZFN	Albumin	AAV	[155]
	CRISPR/Cas9	OTC	AAV	[156]
PKU	ABEs	Pah	AAV	[85]
	CBEs	Pah	AAV	[30]
**viral hepatitis**				
HBV	CRISPR/Cas9	HBV	AAV	[158–161]
	CRISPR/Cas9	HBVS	Lentiviral	[162, 163]
	CRISPR/Cas9	POLK	Lentiviral	[164]
**HCC**	CRISPR/Cas9	G9a	Lentiviral	[166]
	CRISPR/Cas9	ASPH	-	[170]
	CRISPR/Cas9	eEF2k	Electroporation	[167]
	CRISPR/Cas9	NCOA5	Lentiviral	[168]
	CRISPR/Cas9	CXCR4	Electroporation	[169]
	CRISPR/Cas9	CDK7	Lentiviral	[171]
**Ocular disorders**				
RP	CRISPR/Cas9	RHO-P23H gene	Electroporation	[31, 176]
	CRISPR/Cas9	RHO	AAV	[177]
	CRISPR/Cas9	NRL	AAV	[178]
LCA10	CRISPR/Cas9	CEP290	AAV	[32, 174]
LCA1	CRISPR/Cas9	GUCY2D	AAV	[175]
AMD	CRISPR/Cas9	VEGFR2	AAV	[183, 184]
	CRISPR/Cas9	VEGFA or HIFLA	AAV	[185]
	CRISPR/Cas9	VEGFA	LV	[186]
Optic neuropathies	CRISPR/Cas9	pro-degenerative genes in RGCs	AAV	[192]
MECD	CRISPR/Cas9	KRT12	Electroporation	[193]
TGFBICD	CRISPR/Cas9	corneal epithelial reporter	AAV	[194]
**Neuromuscular disorders**				
DMD	CRISPR/Cas9	DMD	AAV	[195]
	ABEs	DMD	AAV	[196]
ALS	CRISPR/Cas9	SOD1	AAV	[34]
	CBEs	SOD1	AAV	[197]
DM1	CRISPR/Cas9	FAH	Adenovirus	[148]
MDC1A	CRISPR/Cas9	HPD	AAV	[198, 199]
**Other genetic diseases make great progress**				
HGPS	ABEs	LMNA	Lentiviral	[76]
	CRISPR/Cas9	TMC1	Cationic lipid	[200]
Genetic deafness	CBEs	TMC1	AAV	[83]

Meesmann's epithelial corneal dystrophy (MECD) is an autosomal dominant disease caused by mutations in the KRT12 gene, which leads to the occurrence of a novel PAM. Researchers designed a CRISPR against mutant single-nucleotide polymorphisms (SNPs) within KRT12 and intrastromal injection was used to deliver the plasmids encoding the CRISPR components to the cornea of a humanized MECD mouse model, successfully editing the mutant KRT12 allele without any off-target effects in the wild-type allele [193]. Another corneal dystrophy named transforming growth factorβ-induced (TGFBI) corneal dystrophy is also a model of autosomal dominant disease that is used to evaluate the effectiveness of CRISPR/Cas9 in two allele-specific systems, contrasting guide-specific cleavage with SNP-derived PAM cleavage [194]. These studies evaluated novel approaches for targeting heterozygous SNPs using CRISPR/Cas9. In addition, optic neuropathies are a group of ON diseases that cause irreversible blindness and are characterized by retinal ganglion cell (RGC) death and ON degeneration. Combining the AAV-mSncg promoter with CRISPR/Cas9 gene editing can knockdown pro-degenerative genes in RGCs and effectively provide neuroprotection in optic neuropathies [192].

#### Neuromuscular disorders

Neuromuscular disorders mainly include Duchenne muscular dystrophy (DMD), limb girdle muscular dystrophies (LGMD), spinal muscular atrophy, Friedreich’s ataxia, Huntington’s disease, and amyotrophic lateral sclerosis (ALS). Gene editing treatment for DMD is one of the most advanced treatments among them. DMD is caused by mutations in a large gene called the dystrophin gene, which leads to the most common large deletions that shift the downstream gene to go out of frame and render the protein product nonfunctional. Moreover, the dystrophin gene cannot be packaged into size-restricted viral delivery vectors because of the vast coding sequence of the dystrophin gene (14 kb). Recently, many works have incorporated the CRISPR/Cas9 system into viral vectors with tropism for skeletal and cardiac muscle with different approaches to explore the therapeutic strategy of DMD and have significantly enhanced the skeletal muscle functions and cardiac hemodynamics in animal models. For instance, studies have focused on alleviating DMD by deleting single or multiple exons [33, 201–206] and performing point mutation repair [207, 208], respectively. In 2016, to correct DMD by skipping mutant dystrophin exons in postnatal muscle tissue *in vivo*, researchers used AAV9 to deliver gene-editing components to DMD model mice, and the dystrophin protein expression in cardiac and skeletal muscle was restored to varying degrees [202]. In the same year, CRISPR/Cas9 system was used in a mouse model of DMD to remove the mutated exon 23 from the dystrophin gene. Exon 23 deletion by CRISPR/Cas9 resulted in the expression of the modified dystrophin gene, and the functions and phenotypes of associated proteins and tissues were partially restored [204]. Researchers also produced specific CRISPR/Cas9-HCAdV to target DMD, and CRISPR/Cas9-HCAdV proved to be efficient in delivering the respective CRISPR/Cas9 expression units and introducing the desired DNA DSBs at intended target sites in immortalized and primary cells [201]. The use of single or dual AAV vector delivery of a muscle-specific Cas9 cassette together with sgRNA cassettes fully corrected the mutation in a dystrophin homology region [205]. Later, DMD model mice were treated intravenously with AAV-mediated CRISPR gene editing and evaluated for disease rescue at 18 months. The nominal dystrophin levels in skeletal muscle and cardiac tissue were restored, but histology and hemodynamics were not improved. The gRNA was found to be depleted, suggesting that gRNA vector loss is a unique barrier for systemic AAV-mediated CRISPR gene editing therapy and that the vector dose needs optimization [33]. In 2018, researchers showed that using CjCas9 as a gene-editing tool to correct an out-of-frame Dmd exon in Dmd knockout mice enhanced muscle strength without off-target mutations [203]. SaCas9 was also proven to have the ability to edit the human DMD gene [206]. Furthermore, BE, as a novel method of gene editing, also applies to the treatment of DMD. In 2018, the split ABE gene was delivered by AAV vectors to muscle cells in a mouse model of DMD to correct nonsense mutations in the Dmd gene, demonstrating the therapeutic potential of BEs in adult animals [196].

ALS is an incurable neurodegenerative disease that usually causes selective loss of motor neurons in the cortex, brain stem, and spinal cord. Because of the diverse genetic origin of ALS, at least 20 genes have been shown to be related to ALS, such as the variants of the SOD1, C9orf72, FUS, and TARDBP genes [209]. Superoxide dismutase 1 (SOD1) mutation is one of the most notable causes [210]. To modify the mutant SOD1 gene, the AAV-SaCas9-sgRNA system was tested to modify mutant SOD1 in SOD1G93A transgenic mice and successfully deleted the SOD1 gene. It was reported that the lifespan of SOD1G93A mice was prolonged by 54.6% [34]. Moreover, the mutations in other genes associated with ALS were corrected by the CRISPR/Cas9 system in animal models and patient-derived iPSCs [209]. BEs have held tremendous potential to treat molecular and genetic diseases since the creation. An intein-mediated trans-splicing system that enables the delivery of CBEs consisting of the widely used SpCas9 protein *in vivo* was engineered. In the G93A-SOD1 mouse model of ALS, intrathecally injected dual AAV particles encoding a split-intein CBE designed to trans-splice and insert a nonsense-coding substitution into a mutant SOD1 gene prolonged survival and noticeably slowed disease development [197]. Studies of other neuromuscular disorders are ongoing as well. For example, myotonic dystrophy type 1(DM1) was treated by CRISPR/Cas system mediated repeat region deletion [211], and muscular dystrophy type 1A (MDC1A) was treated by CRISPR/Cas system-mediated intronic deletion and dCas9 activation in animal models [198, 199] (Table 3).

#### Other diseases

The number of reported *in vivo* clinical trials of CRISPR-based therapeutic gene editing are much fewer than *ex vivo* due to the difficulty of technology and the complexity of the internal environment. In addition to the CRISPR/Cas9-based EDIT-101 drug product for the treatment of LCA10 is in progress of clinical trial phase 2, the treatment of HPV-related cervical intraepithelial neoplasia I, EBT-101 drug product for the treatment of HIV-1 infected adults, NTLA-2001 drug product for the treatment of hereditary transthyretin amyloidosis with polyneuropathy (ATTRv-PN) and with transthyretin amyloidosis-related cardiomyopathy (ATTR-CM) (Table 2) based on TALEN or CRISPR/Cas9 are all on the clinical trials (ClinicalTrials.gov Identifier: NCT03057912, NCT05143307, NCT05144386, NCT04601051). Among them, cervical intraepithelial neoplasia (CIN) and cervical cancer are major causes of persistent HPV infection. E6 and E7 play important roles in HPV-driven carcinogenesis and are appealing therapeutic intervention targets. Previous evidence showed that when HPV16 and HPV18 E6/E7 DNA were disrupted by the designated genome editing tools TALEN and CRISPR/Cas9, the expression of E6/E7 was significantly decreased, inducing cell apoptosis and inhibiting cell line growth. EBT-101 is an HIV-1-specific CRISPR/Cas9 gene editing system delivered by AAV9 for intravenous (IV) administration. Eligible participants received a single IV dose of EBT-101 and were required to attend multiple study visits at irregular intervals for safety monitoring. The duration of the long-term follow-up (LTFU) study will be up to 15 years.

Hutchinson-Gilford progeria syndrome (HGPS) is a rare genetic disease caused by single point mutations. The gene that codes for nuclear lamin A, LMNA, usually contains a dominant-negative C-G-to-T-A mutation (c.1824 C > T; p.G608G). This mutation leads to RNA mis-splicing, which results in progerin, a lethal version of lamin A. Progerin is a toxic protein that induces the premature aging. In 2020, the first HGPS monkey model with typical HGPS phenotypes was generated by microinjecting a BE mRNA and gRNA into monkey zygotes that target the LMNA gene with high success rates [212]. ABEs can convert targeted A·T base pairs to G·C base pairs with few byproducts, without the need for donor DNA templates or DSBs. Injecting ABE-expressing AAV9 at postnatal day 14 directly fix the pathogenic HGPS mutation in a mouse model of HGPS in 2021, this increased the mice's vigor and significantly extended their median longevity from 215 to 510 days [76].

A dominantly or recessive inherited form of genetic deafness caused by the point mutation of transmembrane channel-like 1 gene (TMC1). To maintain normal auditory function, TMC1 encodes a protein that forms mechanosensitive ion channels in sensory hair cells of the inner ear. The point mutation of TMC1 leads to complete loss of auditory sensory transduction. To ameliorate hearing loss in a mouse model, researchers engineered a Cas9-gRNA complex delivered by cationic lipids *in vivo* and found that genome editing agents disrupted the dominant deafness-associated allele in TMC1, reducing progressive hearing loss in 2018 [200]. Later, researchers developed a BE strategy to treat this form of deafness. After testing several optimized CBEs and gRNAs, the most promising CBE derived from an activation-induced cytidine deaminase was chosen and delivered by AAV using a split-intein delivery system, which eventually successfully improved the hearing of the mouse models [83].

### Challenges to clinical translation

Multiple studies have brought tremendous progress in therapeutic genome editing. Nevertheless, the clinical translation of this unique technology still faces many challenges, especially targeting, safety and delivery issues.

#### Ontarget activity maximization

The target sites of CRISPR, BEs and PEs are constrained due to the PAM specificity of Cas proteins. To maximize on-target activity while minimizing unwanted editing, directed evolution and engineered variants of SpCas9 are necessary. For example, in 2020, Walton et al*.* developed a variant named SpG that is able to target an expanded set of NGN PAMs. They further optimized the enzyme, and a near-PAMless SpCas9 variant named SpRY was developed. SpG and SpRY eliminated the NGG PAM requirement [213]. The same year, Shannon et al. reported on the directed evolution of three novel SpCas9 variants that could recognize NRRH, NRTH, and NRCH PAMs (where R is either A or G and H is either A, C, or T), successfully expanding the SpCas9 sequence space that was accessible to PAMs [66]. Hiroshi et al. in 2018 engineered a SpCas9 variant (SpCas9-NG) that can recognize relaxed NG PAMs, extending the recognizable PAM sequence [214]. Kleinstiver et al. in 2015 established two variants of SpCas9 called VQR and VRER, which recognized the novel PAM sequences NGAN/NGNG and NGCG, enhancing the opportunities to utilize SpCas9 in the CRISPR/Cas9 platform [215]. Moreover, researchers also used molecular evolution to modify the NNGRRT PAM specificity of SaCas9 [216–218].

#### Targeting specificity maximization

In addition to improve the targeting scope of CRISPR tools, approaches to maximize targeting specificity and minimize the off-target effects of the CRISPR/Cas9 system are unmet needs. It was possible to try to make efforts in these three aspects: reforming Cas9 variants [219–223], modifying sgRNA [224] and improving the delivery platform of CRISPR/Cas9. For example, SpCas9-HF1 [219], eSpCas9 [220], evoCas9 [221], HypaCas9 [222] and Sniper-Cas9 [223] were engineered to reduce non-specific DNA contacts and all of them maintained robust on-target cleavage. In 2019, Kocak et al. demonstrated that adding a hairpin secondary structure to sgRNAs' spacer region (hp-sgRNAs) can boost the specificity over 55-fold when combined with different CRISPR effectors [224]. Moreover, many other methods of modifying sgRNAs to reduce off-target effects exist: selection [225–227], truncation [228, 229] or extension [230] of guide sequences. To improve the delivery platform of CRISPR/Cas9, Sojung et al. showed that delivering purified Cas9 ribonucleoproteins improved the efficiency of genome editing in human cells in 2014 [231]. Suresh et al. demonstrated that the cell-penetrating peptide-mediated delivery of Cas9 protein and gRNA can effectively reduce off-target effects [232].

Compared to CRISPR/Cas9, BEs and PEs allow targeting specificity with fewer indels and fewer off-target effects. Indels caused by CBE and ABE are 1.1% and 0.1%, respectively, as opposed to the substantially greater 4.3% indels caused by Cas9-HDR editing [53, 54]. Additionally, at four main Cas9 off-target loci, PEs averaged 0.6% off-target alterations as opposed to Cas9 + sgRNA, which averaged 32% off-targeting at the same four loci [22]. To further eliminate the off-target effects of BEs, researchers engineered CBE variants that minimized Cas9-independent off-target DNA editing by approximately 10 to 100-fold [233]. While high-fidelity Cas9 was fused to BE2 and BE3 to develop HF-BE2 and HF-BE3, respectively, aiming to limit the Cas9-dependent off-targeting, the HF-BE2 showed several-fold lower off-targeting, and HF-BE3 showed 37-fold lower off-targeting relative to traditional BEs [234]. Co-expression of free UGI with BE3 containing triple UGI [235] and fusion of bacteriophage Gam protein with BE3 and BE4 [236] are the other two efforts to reduce BE off-targeting.

#### Safety and delivery issues

Regarding the off-target effect, whether it can refer to all the conditions of therapeutic genome editing remains unclear since a therapy always targets one site within billions of DNA base pairs, modifies millions of cells, and varies among patients [219, 220]. Second, the human immune reaction and cytotoxicity are also tricky matters, and how the human immune system will respond to the *in vivo* administration of genome-editing tools and genetically modify cells remains unknown. Viral delivery systems have relatively high efficiency in transgene delivery but are controversial in the latent cytotoxicity they may cause; adenoviral vectors may lead to immune elimination of infected cells [98], and LVs have the risk of potential oncogenesis [115].

## Molecular diagnosis

### Current state of disease diagnosis

Currently, nucleic acid-based diagnostics are the best methods to detect various diseases. The speed and accuracy of disease diagnosis are of vital importance to the prevention and treatment of diseases, especially those caused by infectious viruses. A more recent example is the worldwide pandemic of COVID-19. During the outbreak of COVID-19, the fast and accurate nucleic-acid-based testing is central and essential for controlling the spread of disease, suggesting the need for innovative detection methods with higher sensitivity and specificity.

One of the most required elements for disease detection is nucleic-acid-based biomarkers, which are able to amplify trace amounts of DNA or RNA and then pair complementary nucleotides with high specificity. In addition to disease diagnosis, nucleic acid-based biomarkers are also applied for agriculture, food safety and environmental monitoring. Currently, the most common technique for identifying nucleic acid-based biomarkers is quantitative polymerase chain reaction (qPCR), or sequencing combined to RT in the case of RNA. Because of its versatility, robustness, and sensitivity, it is the gold-standard technique for most nucleic acid-based diagnostics of various diseases. To obtain more reliable and reproducible results, numerous processes need to be optimized, include amplicon detection, primer design, DNA or RNA extraction, and data normalization [237].

However, the process of PCR exhibits nonspecific amplification, which reduces the specificity of detection, even though heat cyclers are not needed for isothermal nucleic acid amplification [238]. Some extra readouts such as fluorescent probes, oligo strand-displacement probes, and molecular beacons, may relatively improve the specificity [239–241], but the costs of reagents, laboratory equipment and trained technicians are high [237]. Therefore, a new detection technique needs to be engineered with the advantages of ease of use and cost efficiency of isothermal amplification with the diagnostic accuracy of PCR.

The new next-generation detection technique is supposed to be single-nucleotide specific. This is required for the identification of the most dangerous pathogenic bacterial or viral variations and strains as well as the detection of genotyping, cancer, and mutations that confer resistance to antibiotics, antiviral medications, or cancer therapies. CRISPR/Cas is widely known for its use as a gene-editing tool. Because of its high specificity to detect DNA and RNA sequences, CRISPR-based diagnostics are able to fulfill these unmet needs, and various CRISPR systems have been modified for nucleic acid detection in recent years.

### CRISPR-based diagnostics

The CRISPR/Cas system is a fundamental part of a prokaryotic adaptive immune system in various archaea and bacteria [45]. It targets foreign genomes based on their sequence and subsequently eliminates them through the endonuclease activity of the Cas enzyme. Diverse Cas enzymes exist among different species of archaea and bacteria and are composed of various CRISPR/Cas systems. The crRNA guides Cas proteins to recognize and cleave nucleic acids that are targeted, and they have high specificity to target specific DNA and RNA sequences, which makes CRISPR/Cas systems have the potential to offer cost-effective, portable and point-of-care diagnosis through nucleic acid screening of diseases.

#### Nucleic acid detection with CRISPR/Cas13

The Cas13 effector is already frequently used to create RNA knockout models, but more recently, it has also been used as a molecular diagnostic tool for accurate and precise RNA detection. The 900–1,300 amino acid CRISPR/Cas type VI (Cas13) family of enzymes is employed to identify ssRNA in the *cis* conformation and exhibits collateral trans-cleavage activity against ssRNA *in vitro* [242, 243].

**Fig. 4:**
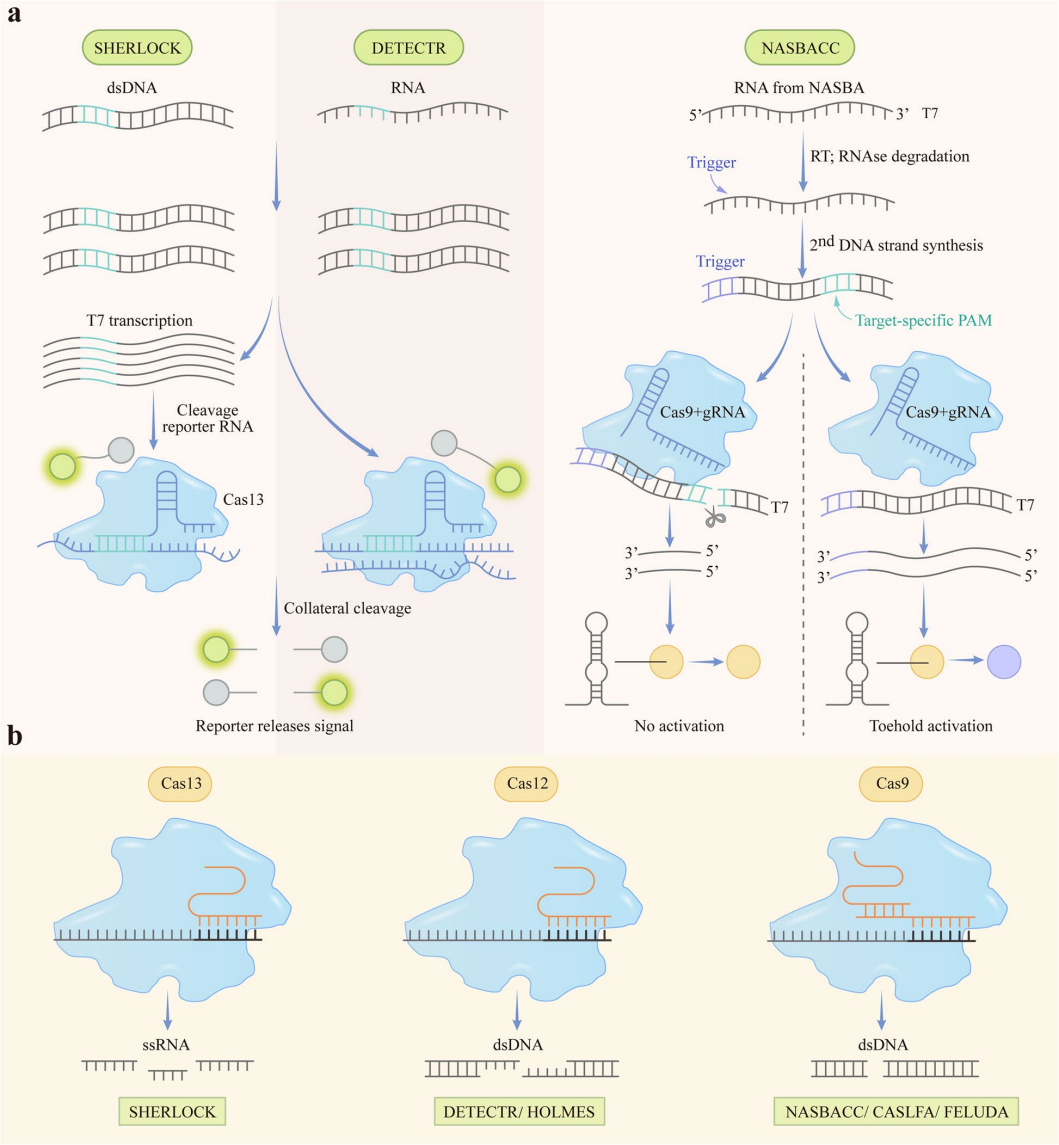
Two strategies for CRISPR-based diagnostics and comparison and detection characteristics of the properties of CRISPR/Cas13/Cas12/Cas9 system. **A** Two strategies for CRISPR-based diagnostics. Left: Schematic of SHERLOCK and DETECTR. DNA or RNA is amplified by RPA or RT-RPA, respectively. T7-transcribed RNA is the amplified product by RPA, and the CRISPR/Cas13 (used in SHERLOCK) and CRISPR/Cas12 (used in DETECTR) systems bind of the crRNA to the complementary target sequence to activate the Cas enzyme and trigger collateral cleavage of quenched fluorescent reporters. Right: Schematic of NASBACC. RNA targets are amplified through NASBA, from reverse transcription (RT) to complementary DNA, a sequence-specific primer that appends a trigger sequence (purple) for the toehold sensor was used. **B** Comparison and detection characteristics of the properties of CRISPR/ Cas13/Cas12/Cas9 system. PAM: protospacer adjacent motif; PFS: protospacer flanking site; DSB: double-strand break

After finishing the study of most of the CRISPR nucleases from microorganisms, Zhang’s group characterized the class 2 type VI CRISPR/Cas effector C2c2 (also known as Cas13a) from the bacterium *Leptotrichia shahii* and demonstrated its RNA-guided ribonuclease function. These findings broaden the range of CRISPR/Cas systems and suggest that Cas13a can be used to develop new RNA-targeting tools [243]. However, the Cas13a was found to have the ability to cut ssRNA randomly and nonspecifically after completing specific RNA cutting, which means that Cas13a has a strong cytotoxicity and cannot be used as a tool for gene editing, such as Cas9. In the same year, Doudna’s group showed that the unique dual RNase activities of Cas13a enable its two distinct catalytic capabilities, multiplexed processing and loading of gRNAs, which in turn allow for sensitive cellular transcript detection. In the reaction system of molecular detection, a ssRNA with chemical modification is used as the substrate. When Cas13a specifically cleaves the target RNA, it can also cleave the ssRNA substrate. The chemically modified ssRNA will not emit fluorescence until it is cut off, and then transcript detection is achieved [242]. Finding a more rapid, cheap and sensitive way to detect pathogens is the goal scientists are striving for. To achieve this goal, Zhang’s group created a SHERLOCK (Specific High Sensitivity Enzymatic Reporter UnLOCKing) detection platform based on CRISPR/Cas13a (Fig. 4a), and they established a CRISPR-based diagnostic (CRISPR-Dx) by combining the collateral effect of Cas13a with isothermal amplification. First, using a forward primer with the addition of a T7 promoter, recombinase polymerase amplification (RPA) or RT-RPA, respectively, isothermally amplifies DNA or RNA. The *Leptotrichia wadeii* Cas13a (LwaCas13a) complex and a crRNA containing the target's complementary sequence bind to the targeted sequence after the T7 promoter permits RNA production of the target. Cas13 is activated and cleaves both the on-target RNA and ssRNA reporter molecules by *cis* cleavage and collateral *trans* cleavage, respectively. The ssRNA reporter molecule is composed of a fluorophore and quencher binding with a short RNA oligomer, and the fluorophore separates from the quencher to produce fluorescence once the oligomer is cleaved. The entire procedure has been demonstrated to successfully detect certain strains of the Zika and Dengue viruses, discriminate pathogens, genotype human DNA, and detect cell-free tumor DNA mutations. It also offers quick DNA or RNA detection with attomolar sensitivity and single-base mismatch specificity [38]. Version two of SHERLOCK (SHERLOCKv2) introduces an immunochromatographic assay-based lateral-flow readout, using antibody-conjugated gold nanoparticles on a paper strip to detect cleaved reporter molecules. In addition, the SHERLOCKv2 has achieved the quantitative multiplexed sensing of nucleic acids and the target detection at zeptomolar (10^–21^ M) concentrations [244].

#### Nucleic acid detection with CRISPR/Cas12

SHERLOCK is a very sensitive and specific tool in the detection of target RNA, but for the detection of target DNA, *in vitro* transcription of DNA to RNA must be conducted before the SHERLOCK test, which is inconvenient. The Cas12 enzyme is an effective tool to achieve the diagnosis of target dsDNA and ssDNA with the requirement of a PAM site in the target region for dsDNA cleavage and the collateral cleavage of ssDNA [245].

Doudna’s group focused on Cas12a, which like CRISPR/Cas9, has the ability to generate targeted DSBs and has been harnessed for genome editing. They found that *Lachnospiraceae bacterium* Cas12a (LbCas12a) or Cas12a from other organisms can facilitate RNA-guided DNA binding, which releases indiscriminate ssDNA cleavage activity. Because Cas12a degrades ssDNA molecules completely with the help of a complementary crRNA that enables Cas12a to be guided to dsDNA. Then, the quencher from the fluorophore is separated as a result of target recognition and reporter cleavage, and a fluorescence signal is produced. Moreover, other type V CRISPR/Cas12 enzymes also have the property of target-activated, nonspecific single-stranded deoxyribonuclease (ssDNase) cleavage. With the help of Cas12a, ssDNase was activated with isothermal amplification, a DNA endonuclease-targeted CRISPR trans reporter (DETECTR) was created (Fig. 4a), and with the combination of RPA preamplification, the sensitivity of DETECTR reached attomolar. DETECTR enabled rapid and specific detection of human papillomavirus in patient samples and achieved DNA detection for the first time [39]. Moreover, the CRISPR-based DETECTR assay provides an alternative to the US Centers for Disease Control and Prevention SARS-CoV-2 real-time RT–PCR assay due to its visual and fast characteristics [37].

Subsequently, Wang’s group used a quenched fluorescent ssDNA reporter as the probe and employed PCR for preamplification together with LbCas12a engineered a one-Hour Low-cost Multipurpose highly Efficient System (HOLMES). HOLMES has the ability to quickly detect both target DNA and RNA. In a reaction system that exists in target DNA, the Cas12a/crRNA binary complex and the target DNA form a ternary complex, transcleaving a nontargeted ssDNA reporter and illuminating fluorescence in the system [41]. Then, in order to identify SNPs and various viruses, such as the Japanese encephalitis virus (JEV) better [246], HOLMES was optimized to HOLMESv2 by using loop-mediated isothermal amplification (LAMP) in conjunction with a thermostable Cas12b from *Alicyclobacillus acidoterrestris* (AacCas12b) in a one-pot reaction [247]. Similarly, the limit of detection (LOD) for HOLMES and HOLMESv2 is roughly 10 aM.

#### Nucleic acid detection with CRISPR/Cas9

In addition to Cas13a and Cas12a, Cas9 combined with other techniques can also be used for the detection of specific DNA and RNA sequences, which again broadens the field of molecular diagnosis. Guide-directed reconstitution of split proteins by catalytically inactive Cas9 partners [248], Cas9-based destruction of PAM-containing sites [249], and Cas9-induced unwinding of the non-targeted DNA strand as a targeting site for isothermal amplification [250] are three main principles of many different Cas9-based approaches for sensing DNA.

In 2016, Collins’s group used a novel CRISPR/Cas9-based module nucleic acid sequence-based amplification (NASBA)-CRISPR cleavage (NASBACC) to detecte clinically relevant concentrations of Zika virus sequences and show selectivity against closely related Dengue virus sequences. Through nucleic acid sequence-based amplification, the amplification of targeted RNA starts with RT to complementary DNA using a sequence-specific primer that appends a trigger sequence (magenta) for the toehold sensor. Then, the RNase H destroys the RNA from the RNA/DNA hybrid, creating the chance for the binding of the primer containing a T7 promoter and producing a complementary second DNA strand. A toehold sensor for the readout and PAM-dependent target detection is attached to nucleic acid sequence-based amplification through RT, and then Cas9 mediates the cleavage in the CRISPR/Cas9-based method (Fig. 4a). When the RNA fragment contains a PAM sequence, Cas9-mediated cleavage produces a truncated RNA that lacks the trigger region for T7 transcription; otherwise, the trigger containing full-length RNA activates the toehold sensor and produces a visible change in color. The technique successfully detected Zika virus (ZIKV) in the low femtomolar range in infected monkey plasma by sensing strain-specific PAM sites [251].

In 2019, Xing’s group developed a novel CRISPR/Cas9-triggered isothermal exponential amplification reaction (CAS-EXPAR) strategy mediated by CRISPR/Cas9 cleavage and nicking endonuclease (NEase)-mediated nucleic acid amplification for site-specific and real-time fluorescent nucleic acid detection [252]. In 2020, Wang’s group improved the efficiency and precision of immune response analytical techniques by introducing the CRISPR/Cas9 system into the lateral flow assay, termed the CRISPR/Cas9-mediated lateral flow nucleic acid assay (CASLFA). CASLFA is able to detect Listeria monocytogenes, genetically modified organisms, and African swine fever virus [253]. In 2021, the FELUDA (FNCAS9 Editor-Linked Uniform Detection Assay) was developed using a catalytically inactive form of Cas9 to identify the targeted mismatches, bind to target DNA, but do not cleave it. The FELUDA was demonstrated to be successfully used for SARS-CoV-2 molecular testing [246].

Compared with CRISPR/Cas9 systems, CRISPR/Cas12 systems and CRISPR/Cas13 systems have the ability to trigger non-specific collateral cleavage on target recognition (Fig. 4b). The cleavage of non-targeted ssDNA by Cas12 and ssRNA by Cas13 are involved in collateral cleavage activity. The collateral cleavage activity provides the detection of nucleic acids by signal amplification and allows for various readouts by the addition of functionalized reporter nucleic acids.

#### Outlook of CRISPR-based molecular diagnosis

In recent years, the field of the CRISPR diagnosis has expanded rapidly, growing from the specific set of molecular biological discoveries to several active clinical trials (ClinicalTrials.gov identifiers: NCT05143593, NCT04535505, NCT04178382, NCT04074369, NCT04535648), multiple COVID-19 tests and the establishment of several companies (Table 4). CRISPR-based detection methods are combined with pre-existing preamplification and readout technologies to achieve a sensitivity and reproducibility equivalent to and comparable to the current first-rate standard nucleic acid detection methods. However, there are still several limitations that need to be optimized to diagnose the diseases and monitor the progression of pathogens. The dependence on preamplification when detecting targets below the femtomolar range is one of the major limitations of most current CRISPR-based diagnostics, which increases the complexity, cost and reaction time. The potential methods to solve this problem may be the incorporation of non-primer-based signal-amplification strategies or modifications of the Cas enzyme, crRNA or reporter molecule. Moreover, sample preparation is another issue that makes the diagnosis process more complex, as it requires a separate step and special heating devices with an incubation program.

**Table 4:** Introduction of reported CRISPR-based diagnostics

Name of diagnostic tools	Enzyme	Assay time (min)	Readout	Applications	Reference
**CRISPR type VI**					
**-**	Cas13	NS	Fluorescence	Detection of human mRNA; detection of bacteriophage λ-RNA	[242, 243]
SHERLOCK	Cas13	132 (NASBA) or 120 (RPA) and 60–180 (CRISPR)	Fluorescence	Detection of viruses (ZIKV, DENV) and bacteria (*E. coli, K. pneumoniae, P. aeruginosa, M. tuberculosis, S. aureus*); discrimination between virus strains; detection of SNPs	[38]
SHERLOCKv2	Cas13	60 (RPA) and 60–180 (CRISPR) or 60–180 (one pot)	Fluorescence or lateral flow	Detection of viruses (ZIKV, DENV) and bacteria (E. coli, K. pneumoniae, P. aeruginosa, M. tuberculosis, S. aureus); discrimination between virus strains; detection of SNPs	[244, 254]
SHINE	Cas13	50 (one pot)	Fluorescence or lateral flow	Detection of SARS-CoV-2	[255]
STOPCovid	Cas12b	60 (one pot)	Fluorescence or lateral flow	Detection of SARS-CoV-2	[256]
CARMEN	Cas13	20 (RPA) and 180 (CRISPR)	Fluorescence	Detection of 169 viruses; subtyping of influenza A	[257]
APC-Cas	Cas13	110 (APC) and 30 (CRISPR)	Fluorescence	Detection of *S. enteritidis*	[258]
	Cas13	< 240	Electrochemical	Detection of microRNAs (miR-19b and miR-20a)	[259]
PECL-CRISPR	Cas13	30 (CRISPR), 30 (phosphorylation of pre-trigger), 30 (EXPAR)	Electrochemiluminescence	Detection of microRNAs (miR-17, let‐7 family miRNAs)	[260]
**CRISPR type V**					
DETECTR	Cas12a	10 (RPA) and 60–120 (CRISPR)	Fluorescence	Detection of HPV16 and HPV18 in human samples	[39]
Cas14-DETECTR	Cas14 (Cas12f)	NS (PCR) and 120 (CRISPR)	Fluorescence	Detection of HERC2 SNPs in human samples	[261]
HOLMES	Cas12a	88 (PCR) and 15 (CRISPR)	Fluorescence	SNP discrimination in cell lines and human samples; detection of viruses (PRV, JEV); virus-strain discrimination	[41, 262]
CRISPR-materials	Cas12a	40 (RPA) and 240 (CRISPR)	Fluorescence or μPAD (visual and electronic)	Detection of EBOV synthetic RNA	[263, 264]
CDetection	Cas12b	10 (RPA) and 60–180 (CRISPR)	Fluorescence	Detection of HPV16; human ABO blood genotyping; BRCA1 and TP53 SNPs	[265]
HOLMESv2	Cas12b	40 (LAMP) and 35 (CRISPR) or 120 (one pot)	Fluorescence	SNP discrimination in cell lines; RNA virus detection (JEV); human mRNA and circular RNA detection; DNA methylation	[266]
E-CRISPR	Cas12a	30–180	Electrochemical	Detection of viruses (HPV16, PB19) and protein (TGF-ß1)	[267]
**CRISPR type II**					
NASBACC	Cas9	120–360 (one pot)	Colometry	Discrimination between African and American ZIKV	[251]
CRISPR-Chip	Cas9	15	Electrochemical	Detection of gDNA from cell lines and DMD patients	[268]
CRISDA	Cas9 nickase	90	Fluorescence	Detection of gDNA; breast-cancer-associated SNPs in cell lines	[269]
FLASH	Cas9	NS	NGS	Detection of gDNA; antimicrobial resistance genes in clinical samples	[270]
CAS-EXPAR	Cas9	60	Fluorescence	Sensing of methylated DNA; *L. monocytogenes* mRNA	[252]
Cas9nAR	Cas9 nickase	60	Fluorescence	Detection of bacteria (*S. typhimurium, E. coli, M. smegmatis, S. erythraea*); detection of *KRAS* SNPs in cell lines	[271]

With the effort to continuously improve the CRISPR-based diagnostic innovations, this new technology will play an even more important role in molecular diagnosis.

## Conclusions

Genome editing has changed the definition of gene and cell therapy and has been a key factor in correcting many molecular and genetic diseases. Compared to the CRISPR/Cas9 system, the BE and PE systems are simpler and more precise, achieving the correction of point mutations in human genetic diseases that account for more than half of all human genetic diseases [90]. Moreover, BEs and PEs can edit the genome without DSBs and are able to edit both dividing and non-dividing cells [272], greatly increasing the efficiency, targeting scope, and purity of the edited products. However, the targeting specificity of genome editing tools needs to be further optimized, and significant safety and delivery issues, such as off-target effects, human immune reactions, cytotoxicity and delivery efficiency, need to be addressed before genome editing can be widely used for treating human diseases.

The feature of high specificity to recognize and cleave target specific DNA and RNA sequences, makes CRISPR/Cas systems have the potential to offer cost-effective, portable and point-of-care diagnosis through nucleic acid screening of diseases. With the successful creation of CRISPR-based molecular diagnosis such as SHERLOCK, DETECTR, HOLMES NASBACC and so on, the possibilities for the application of the CRISPR system have been extended. More importantly, as disease detection technologies continuously improve, the CRISPR system will be a large step to resist to various diseases, especially pandemic viruses worldwide.

## Data Availability

All data generated or analysed during this study are included in this published article (and its supplementary information files).
